# Characterization and phylogeny of fungi isolated from industrial wastewater using multiple genes

**DOI:** 10.1038/s41598-022-05820-9

**Published:** 2022-02-08

**Authors:** Blessing Amaka Ezeonuegbu, Machido Dauda Abdullahi, Clement M. Z. Whong, Japhet Wisdom Sohunago, Hazem S. Kassem, Clement Ameh Yaro, Helal F. Hetta, Gomaa Mostafa-Hedeab, George D. Zouganelis, Gaber El-Saber Batiha

**Affiliations:** 1 Department of Microbiology, Ahmadu Bello University, Zaria, Nigeria; 2 Department of Botany, Ahmadu Bello University, Zaria, Nigeria; 3 Department of Agricultural Extension and Rural Society, College of Food and Agriculture Sciences, King Saud University, Riyadh, 11451 Saudi Arabia; 4 Department of Animal and Environmental Biology, University of Uyo, Uyo, Akwa Ibom State Nigeria; 5 Department of Medical Microbiology and Immunology, Faculty of Medicine, Assiut University, Assiut, 71515 Egypt; 6 Department of Internal Medicine, University of Cincinnati College of Medicine, Cincinnati, OH 45267-0595 USA; 7 Pharmacology Department, Faculty of Medicine, Beni-Suef University, Beni Suef, Egypt; 8 Human Sciences Research Centre, University of Derby, Derby, DE22 1GB UK; 9 Novel Global Community Educational Foundation, Hebersham, NSW 2770 Australia; 10 Department of Pharmacology and Therapeutics, Faculty of Veterinary Medicine, Damanhour University, Damanhour, 22511 AlBeheira Egypt

**Keywords:** Ecology, Microbiology

## Abstract

The aim of this study was the isolation and molecular characterization of fungi from untreated refinery effluent by using multiple conserved genes. The Fungi isolated were characterized based on PCR amplification and genomic sequencing of the internal transcribed spacer region (ITS), partial β-tubulin (BenA), calmodulin (CaM), and RNA polymerase second large subunit (RPB2) genes, along with morphological characterization. The obtained sequences were subjected to BLAST analysis and the corresponding fungal isolates were assigned species names after comparison with representative sequences available in GenBank. Fifteen (15) Fungi species belonging to four genera of *Aspergillus*, *Penicillium*, *Fusarium*, and *Trichoderma* with *Aspergillus* as the predominant genus were identified. Therefore these genes should be used as molecular markers for species level identification of fungi (especially *Aspergillus* and *Penicillium* as proven in this study.

## Introduction

Wastewater from various industries are composed of organic and inorganic complex pollutants including heavy metals, xenobiotics, polyaromatic hydrocarbons (PAHs), strong acids and suspended materials which cause major environmental pollution worldwide^1,2^. The microorganisms present in wastewater are archaea, bacteria, fungi, algae, protozoa and viruses^3,4^. The abundance and diversity of these organisms are influenced by parameters like temperature, pH, salinity and dissolved oxygen^1^. A great number of molecular studies have investigated archaea and bacterial diversity of various wastewater^5,6^, but fewer have addressed fungi^1^.

Fungi are diverse group of eukaryotic organisms characterized as heterotrophic, saprophytic, symbiotic and parasitic due to their achlorophyllous nature. Their cell walls are made up of β-glucans and chitin^7^. They are known as the second largest after kingdom Animalia with estimate of over 5 million species^8^. Fungi have the ability to metabolically utilize various substrates such as carbohydrates, proteins, lipids, aromatic hydrocarbons, and other chemical compounds as sole sources of carbon^6,13^. They perform several functions in wastewater systems harboring them, which include detoxification, biodegradation and decolourization of pollutants^1,9^.

Apart from known enormous importance of fungi, the taxonomy of these organisms is still challenging due to a lack of reliable and advanced techniques for their identification and systematic studies. Earlier studies on the composition of fungi in wastewater were either dependent on traditional method of identification based on growth, morphology, metabolism and enzymatic activity^1,10^, or the use of one molecular marker gene to identify the fungi to specie levels^11,12^. However, the use of multiple marker genes for identification of fungal communities in wastewater has not been extensively studied. In order to resolve the difficulties of fungal identification to species level, several genetic markers for rapid classification of fungi having conserved sequences, include internal transcribed spacer regions (ITS), Beta-tubulin genes (Ben A), Calmodulin (CaM) and RNA polymerase II gene (RPB2)^8,13–15^.

The internal transcribed spacer regions (ITS) are used as official universal DNA barcode for fungi^14,15^. The ITS1, ITS2, and ITS4 have been proven to be useful for the identification of yeasts and some fungi such *Aspergillus*, *Penicillium*, *Talaromyces*, *Cryptococcus*, *candida*, and *Trichosporon* species among many others^14–16^. However, ITS sequences cannot be used for phylogenetic analyses of unrelated taxa due to low variability and slow evolution. Also, ITS sequences do not always allow correct species differentiation especially among *Aspergillus* and *Penicillium* genera^17,18^. Hence, additional gene markers are essential for correct species delineation. Secondary molecular markers such as beta tubulin; calmodulin and RPB2 have been successfully used in fungal genomics^8,15,18^. Reports have shown that these protein-encoding genes contained highly variable intron regions which contain highly variable introns that evolve at a faster rate compared to ITS^18,19^.

Beta-tubulin genes are found in all eukaryotes encoding for polypeptide proteins. They have been used for phylogenetic analysis in fungi from the entire kingdom to the species level. Four Beta tubulin genes are found in all fungi; two α-tubulin (tub A) and two β-tubulin (tub B) genes. Tub A is responsible for the production of two alpha tubulin polypeptides (alpha 1 and alpha 2) while Tub B produces one alpha polypeptide (alpha 2)^13^. Reports have it that beta-tubulin gene sequences contain 3.5-fold more phylogenetic information than the small sub-unit (SSU) rRNA gene, thus it has been reported that it is an ideal marker for analysis of deep-level phylogenies and for complex species groups^8,18^.

Calmodulin (CaM) is a small acidic protein present in all eukaryotic cells and shown to be highly conserved both functionally and structurally^20,21^. Its primary role is to serve as an intracellular Ca^2+^ receptor which signal proliferation, motility, and cell cyclic progression. Ca^2+^-CaM complexes act by controlling the activity of numerous intracellular proteins such as phosphodiesterase, Ca^2+^-ATPase, serine protein kinases, and protein phosphatases. It also acts on several metabolic pathways and gene expression regulation in many eukaryotic organisms including fungi^20^.

RNA polymerase II gene (RPB2) encodes for second largest protein subunit in eukaryotes which synthesizes mRNA precursors and functional non-coding RNAs^22,23^. A study^24^ reported that RPB2 gene is a viable alternative molecular marker for the analysis of environmental fungal communities due its discriminative power, quantitative representation of community composition and suitability for phylogenetic analyses. Therefore this study was aimed at isolation and molecular identification of indigenous fungi from untreated refinery wastewater using multiple genes.

## Materials and methods

### Collection of untreated refinery effluents

Samples of untreated effluent were collected from waste water channel of Kaduna Refinery and Petrochemical Company (KRPC), Kaduna State, Nigeria. The samples were collected in sterile sample bottles. The bodies of the bottles were rinsed thoroughly with sterile distilled water before transporting them in ice box to the laboratory for fungal isolation^25^.

### Isolation and molecular characterization of test fungi from untreated refinery effluent

#### Isolation of fungi from untreated waste water

The effluent samples were removed from the ice box and kept to stand on a sterile laboratory work bench. 10 ml of the samples in duplicates were aseptically dispensed in sterile centrifuge tubes and centrifuged at a speed of 250 rpm for 10 min to concentrate the samples. A portion (0.1 ml) of the residue of each sample was spread-plated on sterile potato dextrose agar (PDA) (Oxoid ltd, Basinstoke, United Kingdom) and Malt Extract Agar (MEA) (Oxoid ltd, Basinstoke, United Kingdom) plates in duplicate (containing 50 µg/L of chloramphenicol), using sterile bent glass rod. The plates were incubated at room temperature (30 °C) for 7days^26^.

#### Colony morphology and microscopic characterization of fungal Isolates

Colonies grown on each medium were distinguished on the basis of their surface characteristics such as texture, colour, zonation, sporulation and diameters^25^. The distinguishable colonies were sub-cultured on PDA slant and incubated at room temperature (30 °C) for 7 days to obtain pure isolates. The microscopic characteristics were carried out by mounting small portion of the growing region of the fungi on a clean grease free slide with a drop of lacto phenol cotton blue, covered with a cover slip and examined under electron microscope using × 40 objective lens. The isolates were characterized and identified using taxonomic guide^19,27,28^. The pure isolates were maintained in PDA slants and stored in refrigerator for further identification.

#### Molecular identification of fungal isolates

##### Extraction of fungal genomic DNA

Each of the isolates was grown on potato dextrose agar at room temperature for 5 days. This was followed by sub-culturing each isolates into a 250 mL Erlenmeyer flask containing 100 mL potato dextrose broth (Oxoid ltd, Basinstoke, United Kingdom) and incubated for 5 days. The mycelial mass produced by each isolate was separated from the broth by filtration through sterile No. 5 Whatman filter paper. The mycelial mass was crushed using porcelain mortar and transferred to Eppendorf tubes for extraction.

The genomic DNA extraction was carried out using ZR Fungal/Bacterial DNA MiniPrep Kit (Zymo Research, Irvine, CA, USA) according to manufacturer’s manual instructions^14,16,29,30^.

##### PCR amplification of the target genes

Primers specific for internal transcribed spacer region (ITS), beta-tubulin gene (benA), calmodulin gene (CaM) and RNA polymerase II second largest subunit (RPB2) loci are presented in Table 1.

**Table 1:** Primers used for the amplification of specific genes in the fungal isolates.

Locus	Primer	Direction	Oligonucleotide sequence (5′–3′)	Length (bp)	References
Internal Transcribed Spacer (ITS)	ITS1	Forward	TCC GTA GGT GAA CCT GCG G	600	^14,17^
	ITS4	Reverse	TCC TCC GCT TAT TGA TAT GC		
β-tubulin (BenA)	Bt2a	Forward	GGT AAC CAA ATC GGT GCT GCT TTC	550	^14,17,31^
	Bt2b	Reverse	ACC CTC AGT GTA GTG ACC CTT GGC		
Calmodulin (CaM)	CMD5	Forward	CCG AGT ACA AGG ARG CCT TC	580	^14,17^
	CMD6	Reverse	CCG ATR GAG GTC ATR ACG TGG		
RNA polymerase II second largestsubunit (RPB2-1)	5F	Forward	GAY GAY MGW GAT CAY TTY GG	700	^14,17,31^
	7CR	Reverse	CCC ATR GCT TGY TTR CCC AT		

PCR amplification of the extracted DNA was performed in a 20 µL reaction mixture as follow: 1 µL gDNA template, 0.2 µL DNA polymerase, 0.5 µL each forward and reverse primers, 1µL dNTPs and sterile double distilled water to a final volume of 20 µL. The thermocycler was programed for the following PCR conditions: initial denaturation at 94 °C for 5 min, 35 cycles of denaturation at 94 °C for 45 s, annealing at 55 °C for 45 s, and extension at 72 °C for 1 min, with a final extension at 72 °C for 10 min. For the amplification of RPB2 gene region, touch-up PCR conditions of 5 cycles with annealing temperature 48 °C followed by 5 cycles at 50 °C and final 25 cycles at 52 °C were used. After complete amplification, the PCR products were analyzed for gel electrophoresis by using 1% agarose gel (1 g of agarose in 100 ml of Tris buffer) with ethidium bromide as the staining agent^31^.

### Sequencing and phylogenetic analysis

The fungal isolates were identified by DNA sequencing according to standard protocols. Sequencing was carried out in a 28 μl reaction mixture as follows: 4 μl of each primer, 8 μl of purified DNA and 16 μl of PCR water and the samples was sequenced with the Di- Deoxy Terminator sequencer. The contigs (formed from forward and reverse sequences) obtained were analyzed using BioEdit 7.2.5 software and aligned using Clustal W of MEGA 7.0 software^14,32,33^. The fungal isolates were assigned species names after comparison with representative sequences available in NCBI (National Center for Biotechnology Information). The obtained sequences were deposited in GenBank and accession number assigned.

The evolutionary history of the fungi was analyzed using the Maximum Likelihood (ML) method based on the Tamura-Nei model of MEGA 7^33,34^. The bootstrap tree formed from 1000 replicates represents the evolutionary history of the taxa analyzed. The percentage taxa clustered together in the bootstrap test (1000 replicates) are shown next to the branches^31^.

## Results and discussions

### Cultural and microscopic characteristics of fungal isolates

**Figure 1:**
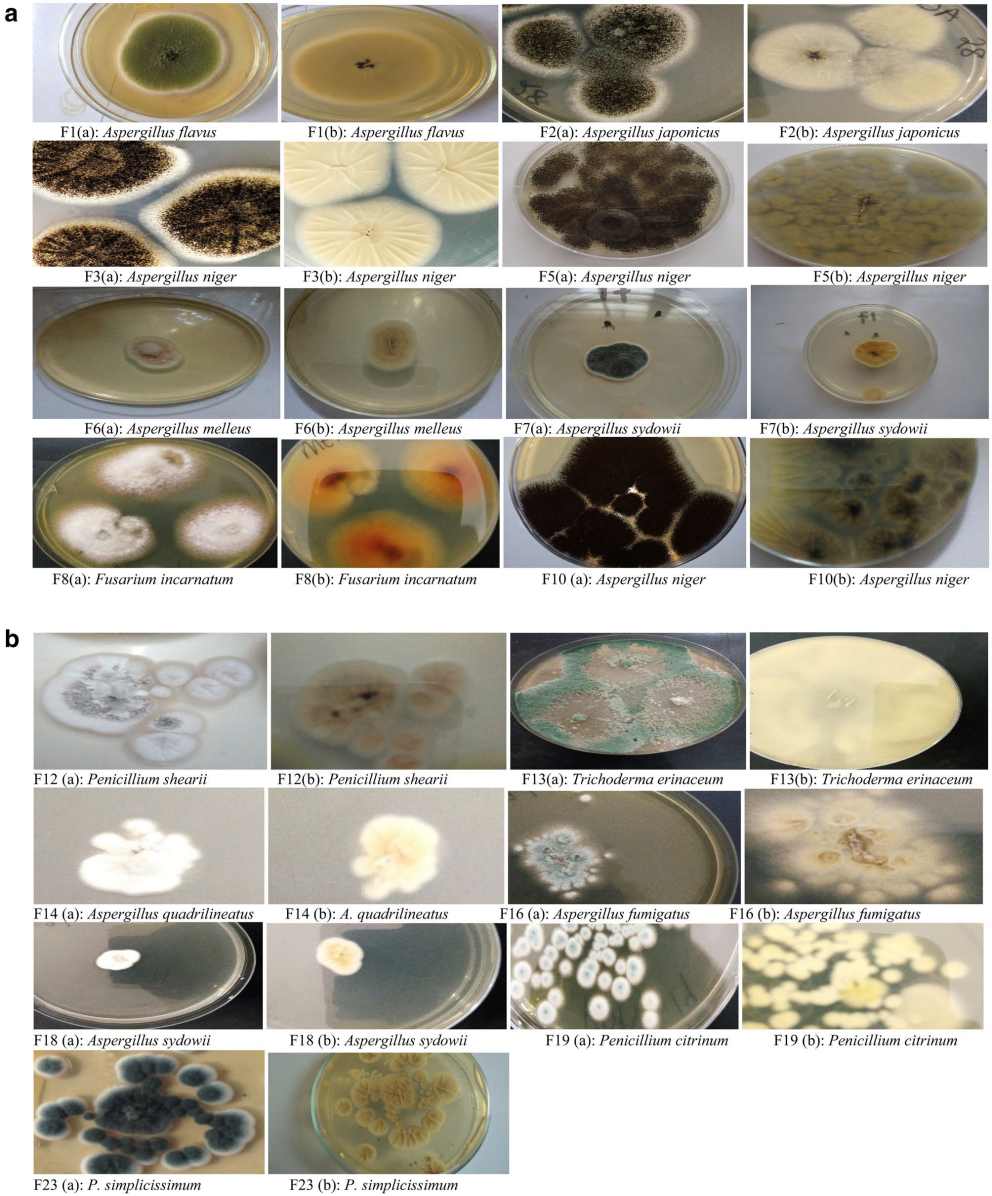
(**a**) Cultural pictures of fungal isolates from refinery effluent. Keys: (a) = Surface characteristics; (b) = reverse characteristics. (**b**) Cultural pictures of fungal isolates from refinery effluent. Keys: (a) = Surface characteristics; (b) = reverse characteristics.

Fifteen (15) fungal isolates consisting of four genera; *Aspergillus*, *Penicillium*, *Fusarium*, and *Trichoderma* were obtained in this study with *Aspergillus* as the predominant genus (Table 2).The results in Table 2 also revealed the cultural features of the isolates (F1 to F23) in terms of colour, surface characteristics, reverse, edge and diameter. Pictorial representations of the surface and reverse characteristics of the fungal isolates are shown in Fig. 1(a) and (b).

**Table 2:** Cultural characteristics of fungal isolates from untreated refinery effluent.

Isolate code	Colour	Surface characteristics	Edge	Reverse colour	Colony diameter mm) (mean ± SD)	Identity of isolates
F1	Mint green	Powdery	White, circular	Cream	2.70 ± 0.00	*Aspergillus flavus*
F2	Black	Granular	White, irregular	Cream	2.50 ± 1.02	*Aspergillus japonicus*
F3	Brownish-black	Black	grey, irregular	Black	2.70 ± 0.14	*Aspergillus niger*
F5	Black	Granular	Black, irregular	Cream	1.90 ± 1.02	*Aspergillus niger*
F6	Pale pink	Granular	light pink, irregular	White	2.35 ± 0.07	*Aspergillus melleus*
F7	Dark-green	Cottony	White, irregular	White	1.35 ± 0.14	*Aspergillus sydowii*
F8	White	Smooth	White, circular	Cream	1.20 ± 0.28	*Fusarium incarnatum*
F10	Black	Granular	White, irregular	Cream	2.80 ± 0.97	*Aspergillus niger*
F12	Whitish gray	Smooth	White, circular	Cream	0.90 ± 0.00	*Penicillium shearii*
F13	Whitish-green	Granular	circular	White	8.00 ± 0.00	*Trichoderma erinaceum*
F14	White	Smooth	White irregular	Cream	1.80 ± 0.00	*Aspergillus quadrilineatus*
F16	Greenish blue	Smooth	White, circular	White	1.15 ± 0.07	*Aspergillus fumigatus*
F18	White	Cottony	White, irregular	Cream	1.85 ± 0.07	*Aspergillus sydowii*
F19	Bluish-green	Cottony	White, irregular	White	1.30 ± 0.00	*Penicillium citrinum*
F23	Dark green	Cottony	White, irregular	White	1.25 ± 0.07	*Penicillium simplicissimum*

**Figure 2:**
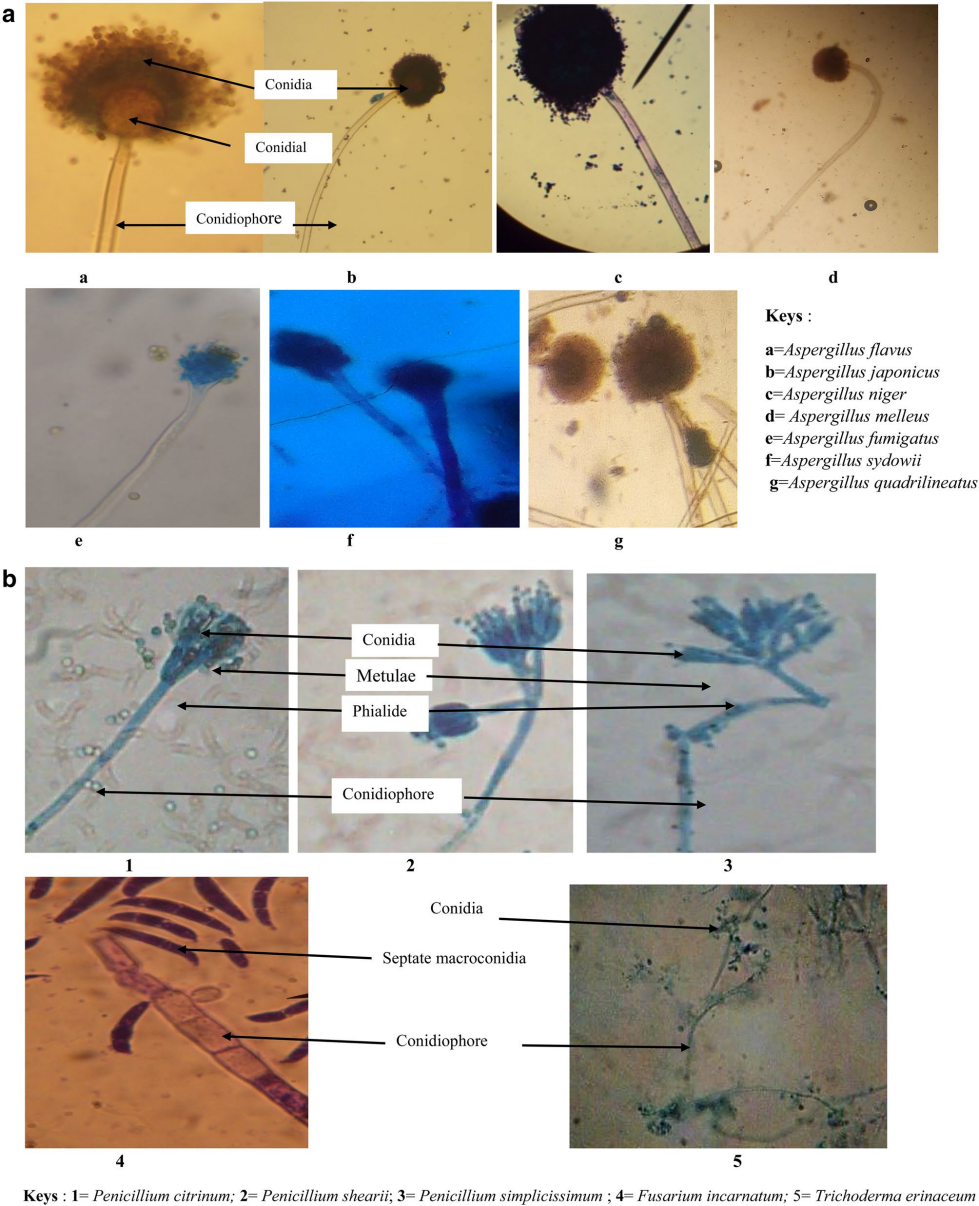
(**a**) Microscopic features of *Aspergillus* species (× 40 magnification). (**b**) Microscopic features of *Penicillium, Fusarium* and *Trichoderma* species (× 40 magnification).

The microscopic features of the isolates are presented in Fig. 2(a) and (b) showing the conidia, spores and conidiophores. *Aspergillus* species had septate hyphae, hyaline conidiophores and radial conidial head bearing the spores (Fig. 2a). *Penicillium* species appeared as septate hyphae with conidiophores and secondary branches (metulae). The metulae bear flasked shaped phialides with unbranched chains of round conidia (Fig. 2b). *Fusarium* species showed septate hyphae, multiseptate canoe shaped macroconidia attached to the conidiophores (Fig. 2b). *Trichoderma* species appeared as septate hyphae, short conidiophores which are flask shaped clustering together at the end of each phialides (Fig. 2b).

The genus *Aspergillus* is one of the most well researched fungi genera with over 200 officially recognized species^35^. The ubiquitous nature of *Aspergillus* may be due to their saprophytic feeding habit as well as their ability to grow in a wide range of environment^25^. This observation sturdily indicates that members of these fungal genera isolated, have the capacity to survive and withstand toxic effects of polycyclic aromatic hydrocarbons^26^.

### Polymerase Chain Reaction (PCR) of fungal isolates obtained

PCR amplification of internal transcribed spacer (ITS), beta tubulin gene (Ben A), calmodulin gene (CMD) and RNA Polymerase II Second Largest Subunit (RPB2) genes of the fungal isolates are discussed below.

#### Amplification of internal transcribed spacer

Thirteen out of the fifteen fungal isolates were positive to PCR amplification of ITS regions, with amplicon sizes of 600 base pairs. Although, the ITS region is widely used as universal primers for fungi, it is not sufficient for identifying most fungi to specie level due to their low variability and slow evolution^17,36^. Visagie et al.^18^ however suggested the use of other molecular markers for accurate identification of fungal species and phylogenetic relationships. Other secondary identification markers for *Aspergillus* and *Penicillium* species (and other ascomycetes) used in this study were beta tubulin; calmodulin and RPB2. These protein-encoding genes contained highly variable intron regions^14,19,37^.

#### Amplification of beta-tubulin gene

Thirteen isolates (13) out of the fifteen (15) isolates were positive with amplicon sizes of 480-600 bp. This results were similar to those obtained in previous studies^38–40^. Eulalia et al*.*^39^ and Kamarudin and Zakaria^40^ amplified *Aspergillus* fragments of beta tubulin genes with amplicon sizes in the range of 550 to 600 bp. Samson et al.^41^ and Erika et al.^42^, obtained beta tubulin gene amplicon sizes of ranging from 432 to 550 bp for *Aspergillus, Penicillium* and other fungal species. Beta-tubulin genes are found in all eukaryotes and have been used for phylogenetic analysis in fungi from kingdom to the species level. Reports have shown that beta tubulin genes have more variability compared to the ITS region^18^. This amount of variation is suitable for determining phylogenetic relationship of closely related species of *Penicillium* and *Aspergillus* genera^19^.

#### Amplification of RPB2 gene

The amplified partial RPB2 genes of the isolates revealed that only two isolates, *P. citrinum* (F19) and *P. citrinum* (F19D) were positive with amplicon sizes of approximately 650 and 600 base pairs respectively. This result is in agreement with the studies of Houbraken & Samson^17^ who identified *Penicillium citrinum* using RPB2 genes.

#### Amplification of Calmodulin gene

The result of the amplified calmodulin genes of the isolates showed that *A. niger* (F5), *A. niger* (F10) and *P. citrinum* (F19) had sizes of 500 bp, 550 bp and 500 bp respectively. Calmodulin gene has been considered important for the identification of *Aspergillus* species, and some reports have even stated it should be used as the primary gene for identification of *Aspergillus* species^38,41^.

### Gene Sequences of Fungal Isolates

Identifications based on cultural features were confirmed by sequence analysis of the isolates. Basic Logical Alignment Search Tool (BLAST) results of ITS region, Beta-tubulin, RPB2 gene and calmodulin gene sequences of this study in National Center for Biotechnology Information (NCBI) provided relationships and similarities with reference sequences in GenBank. The amplified sequences of each gene were submitted to GenBank and their accession numbers were assigned (Table 3). The results in Table 3 revealed that most isolates had above 96% similar identity to reference sequences of GenBank.

**Table 3:** Accession numbers of amplified nucleotide sequences from fungal. Isolates.

Fungi	Isolate	ITS		Beta-tubulin		Calmodulin		RPB2	
		Identity (%)	Accession No.	Identity (%)	Accession No.	Identity (%)	Accession No.	Identity (%)	Accession No.
*Aspergillus flavus*	F1	96.89	MK828704	100	MH180047	–	–	–	–
*A. flavus*	F1D	–	–	100	MG517775	–	–	–	–
*A. japonicus*	F2	99.11	MK840963	100	MH208743	–	–	–	–
*A. japonicus*	F2D	97.53	MK840964	–	–	–	–	–	–
*A. niger*	F3	100	MK828713	99.79	HQ632731	–	–	–	–
*A. niger*	F3D	–	–	100	MH781323	–	–	–	–
*A. niger*	F5	98.99	MK840965	100	MH781323	98.48	JX500080	–	–
*A. niger*	F5D	97.75	MK840966	99.59	LC389053	98.87	MG991517	–	–
*A. melleus*	F6	96.71	MK840967	–	–	–	–	–	–
*A. sydowii*	F7	98.95	MK828705	100	MH426599	96.86	LN898812	–	–
*A. sydowii*	F7D	99.45	MK828710	100	MH644075	96.63	LN898808	–	–
*Fusariumincarnatum*	F8	–	–	98.83	KT374271	–	–	–	–
*F. incarnatum*	F8D	–	–	98.90	KJ020856	–	–	–	–
*A. niger*	F10	99.41	MK828708	100	MH781319	–	–	–	–
*A. niger*	F10D	–	–	100	MH208814	–	–	–	–
*A. fumigatus*	F11	99.27	MK816855	–	–	–	–	–	–
*Penicilliumshearii*	F12	98.96	MK840968	–	–	–	–	–	–
*P. shearii*	F12D	95.83	MK828709	–	–	–	–	–	–
*T. erinaceum*	F13	98.53	MK840969	–	–	–	–	–	–
*A. quadrilineatus*	F14	97.98	MK840970	–	–	–	–	–	–
*A. fumigatus*	F16	–	–	100	MH781343	–	–	–	–
*A. fumigatus*	F16D	–	–	100	MH781334	–	–	–	–
*A. sydowii*	F18	99.41	MK828707	100	LC367596	–	–	–	–
*A. sydowii*	F18D	97.34	MK828706	97.34	MK828706	–	–	–	–
*P. citrinum*	F19	99.27	MK828711	99.63	MG991339	–	–	99.27	MK828711
*P. citrinum*	F19D	99.54	MK840969	–	–	–	–	99.27	MK828711
*P. simplicissimum*	F23	98.93	MK840973	99.02	GU981631	–	–	–	–
*P. simplicissimum*	F23D	99.27	MK828712	99.32	GU981632	–	–	–	–

“–” denotes no clear PCR products were obtained using primers from Table 1.

There has been little or no extensive research on identification of the Fungiusing different molecular marker approach in Nigeria. Focus has been on macroscopic and microscopic features.

### Phylogenetic Tree

**Figure 3:**
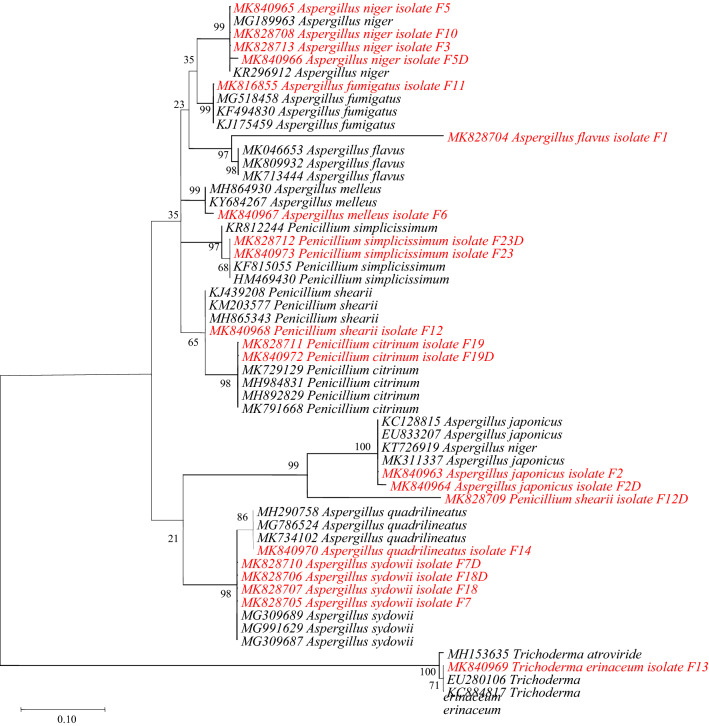
Phylogenetic tree of partial ITS gene sequences by maximum likelihood. Note: Sequences from this study are shown in red.

Phylogenetic trees of the fungal isolates revealed that the isolates were clustered in grouping patterns of close resemblance. Sequences from this study are shown in red colours while sequences from GenBank are shown in black. Test of phylogeny was bootstrap of 1000 replications. Phylogenetic tree based on ITS gene revealed that the alignment matrix contained 54 nucleotide sequences with 209 positions in the final dataset. All isolates of *Aspergillus* and *Penicillium* species were clustered had cluster identity of above 95% with those from GenBank. The tree was out grouped by *T. erinaceum* (Fig. 3).

**Figure 4:**
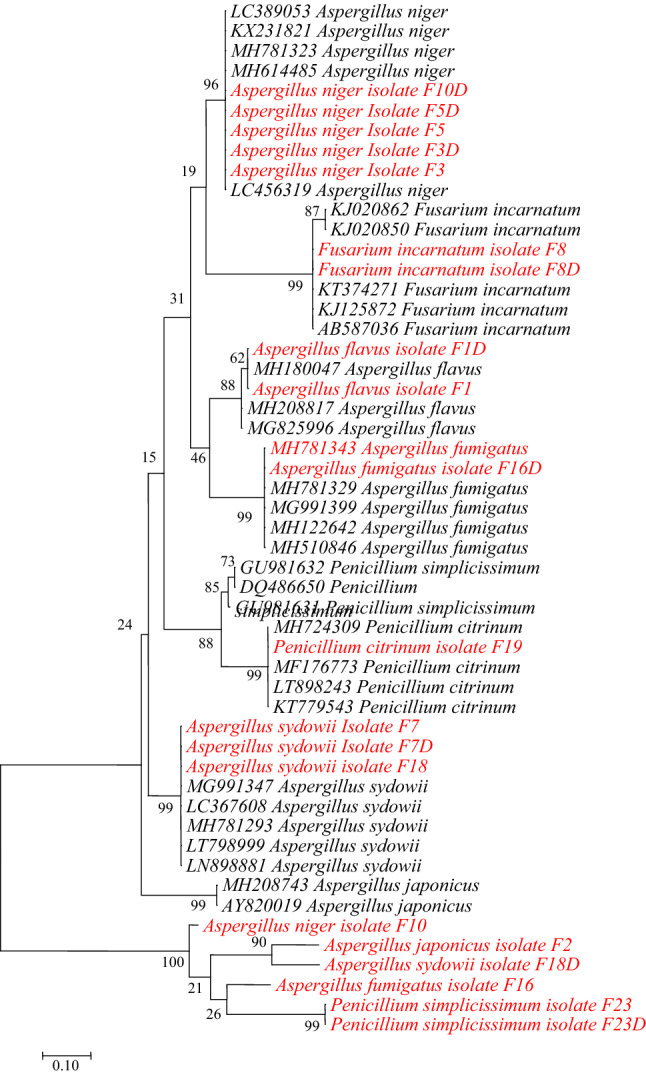
Phylogenetic tree of partial β-tubulin gene sequences by maximum likelihood. Note: Sequences from this study are shown in red.

Beta-tubulin gene alignment matrix contained 52 nucleotide sequences with 19 positions in the final dataset. All the fungal species had above 85% cluster similarity with fungal species from GenBank while *P. Simplicissimum* was placed in the out group (Fig. 4).

**Figure 5:**
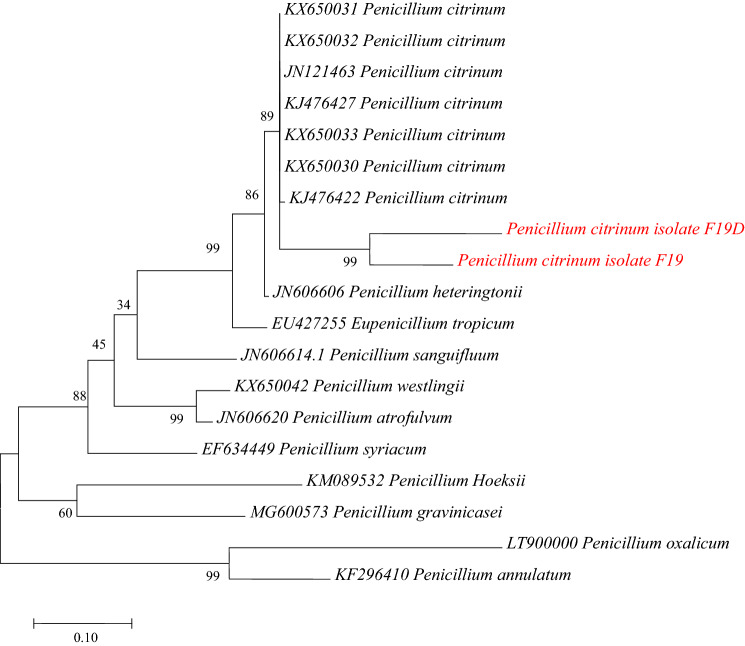
Phylogenetic tree of partial RPB2 gene sequences by maximum likelihood. Note: Sequences from this study are shown in red.

Phylogenetic tree based on partial RPB2 gene revealed that the alignment matrix involved 19 nucleotide sequences with a total of 404 positions in the final dataset. The two positive isolates of *Penicillium citrinum* shared 90% cluster similarities with sequences from GenBank (Fig. 5).

**Figure 6:**
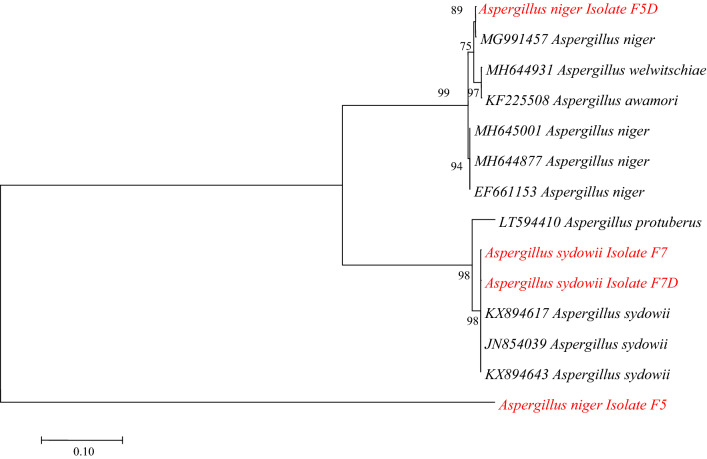
Phylogenetic tree of calmodulin gene sequences by maximum likelihood.

Phylogenetic tree based on calmodulin gene had an alignment matrix of 14 sequences. *A. niger* (F5D) shared 89% cluster similarity while the two isolates of *A. sydowii* (F7 and F7D) had equal (98%) cluster similarities with sequences from GenBank. *A. niger* (F5) falls in the outgroup (Fig. 6).

The phylogenetic trees revealed that related species are clustered together which indicates a clear and well resolved classification and evolutionary history of the isolates^19,37,40^.

## Conclusion

There has been little or no extensive research on identification of the Fungi using different molecular marker approach in Nigeria. Focus has been on cultural and microscopic features. The fungal isolates from this study were further subjected to PCR amplification coupled with DNA sequencing of four molecular genes markers. The fungal species isolated from untreated refinery effluent consist of the following genera; *Aspergillus*, *Penicillium*, *Fusarium*, and *Trichoderma* with *Aspergillus* being the predominant genus. Sequence results obtained revealed above 95% similarities between the isolates in this study and those found in GenBank. The identification and molecular characterization of the fungal isolates to specie level gave a better result by PCR amplification and sequencing of ITS region, partial beta tubulin, calmodulin and RPB2 genes. Therefore should be used as molecular markers for species level identification of fungi (especially *Aspergillus* and *Penicillium* as proved in this study).
